# Registration Difficulties—I

**Published:** 1908-11-07

**Authors:** 


### Nursing Administration.

### REGISTRATION DIFFICULTIES—I.

Few subjects have suffered more from indistinct methods of thought than the proposal for a State registration of nurses. It is seldom that either the opponents or adherents of registration take the trouble to reason out clearly their own attitude towards it, and in consequence a great deal of transparent nonsense is talked on both sides. Recently a leading member of the nursing profession, whose skill in organisation has become almost world-renowned, wrote to the *Times* to repudiate any form of registration which was not universal and compulsory, apparently unaware that the monstrous chimera of penalising women who should tend the sick without a mandate from the State had been long cherished by the present promoters of legislation, and had dissolved like dew before the practical difficulty of getting it even presented to Parliament. The question before the nursing world is the State registration of nurses who desire to be registered, and, however ardently a certain small section of nurses may desire a stringent root and branch measure, it is this voluntary form of registration alone which will ever be entertained. The objections to the formation of a State register on which nurses shall be invited to inscribe their names fall into two divisions, theoretical and practical. The opposition to registration *per se* rests on the contention that the nurse's qualifications for her work are not such as can be gauged by examination. It is a contention which deserves more attention than it has received. There are hundreds, possibly thousands, of excellent nurses practising to-day who could not be got through any examination worthy of the name to save their lives. What would happen if registration became so universal that no nurses whose names were not on the register would have a reasonable chance of gaining their living? It would mean that the women who could not pass examinations or thought they could not do so, whether from temperament or from lack of intellectual gifts would refrain from offering themselves for training. Among these are to be found many of the nurses most acceptable, not only to their patients but also to the medical men under whom they work. Are we under cover of conferring a benefit on the public to sweep away the very nurses whom sick people choose before all others to have at their bedside? Many of the wisest authorities at the Universities are beginning to perceive that the examination test is already applied in far too many cases, and fails under countless circumstances in picking out the best men and women for specified employments. An Appointments Board has even been formed to pick out candidates for certain services irrespective of examination marks, and the doctrine that the man is worth more than the examinee is spreading rapidly. Candidates for the Navy are now selected, not by their precocity in grappling with Greek roots or abstruse mathematical problems, but on the report of the masters who have watched their progress, and with some relation to their disposition and general fitness. While these changes are being made in the right direction in the public services, why is the nursing profession to be hampered with the outworn fetters which the schools are gladly discarding? It is extraordinary that people should still cling to the fancy that the practical ability to perform certain duties is necessarily combined with the ability to explain how and why it is done. By laying the principal stress on the power of passing examinations in the nurse's training, registration would inevitably discourage that natural facility and tenderness in dealing with the sick from high motives which is known as vocation. It is not that the examiners would desire to do so, in all probability they would deal as gently as they could with the candidates, but try as they might, the qualities for which no marks were allotted, the qualites none can gauge save only those under whom the nurse has worked, would go to the wall. It is matter of history that the cause of administrative reform in Egypt was sensibly retarded by attempts to choose men for the work by the old process of examination, until at length Lord Cromer spoke out boldly and declared it was character and temperament which Egypt needed, not mere book learning. If there were ever a profession needing first of all character and temperament it is nursing. It is a work which stands in a peculiar position compared with other callings. It is linked indissolubly with the doctor's work, and cannot stand or fall by itself. It requires a combination of qualities and perceptions. It requires facility in performing duties which could never be tested by examination. It requires tact in intercourse with patient, medical authority, patients' friends. Indisputably the woman whose name appeared at the top of the examination list might be unfitted in every conceivable way, save by ordinary "cram," for entering a sick-room and bearing the responsibilities of life and death. Are not these facts well known to those debating the vital question whether the nurse's fitness is to be determined in the examination room?

It is to be regretted that during the consideration of the Nurses' Registration Bill on Report in the House of Lords, on Monday, this aspect of the question was not brought forward. It was assumed without debate that examinations were the only possible tests of the nurse's qualifications, and should the Bill become law the barrier of examination will stand from henceforth at the door of a profession least of any likely to be benefited by the exclusion from its ranks of indifferent examination subjects, and demanding gifts of a nature impossible to be tested on paper. It is one thing to test nurses systematically by outside examiners in the familiar surroundings of their own institution, taking the result into account when the certificate is granted, but always laying particular stress on the habitual fulfilment of duty in the wards. It is quite another thing to admit or reject women as nurses on the result of their ability to answer during an hour of intense excitement questions adapted solely to test memory and facility for expression.

